# An information-flow-based model with dissipation, saturation and direction for active pathway inference

**DOI:** 10.1186/1752-0509-4-72

**Published:** 2010-05-27

**Authors:** Xianwen Ren, Xiaobo Zhou, Ling-Yun Wu, Xiang-Sun Zhang

**Affiliations:** 1 Academy of Mathematics and Systems Science, Chinese Academy of Sciences, 100190, Beijing, China; 2 Center for Biotechnology and Informatics, The Methodist Hospital Research Institute and Department of Radiology, The Methodist Hospital, Weill Cornell Medical College, Houston, TX 77030, USA

## Abstract

**Background:**

Biological systems process the genetic information and environmental signals through pathways. How to map the pathways systematically and efficiently from high-throughput genomic and proteomic data is a challenging open problem. Previous methods design different heuristics but do not describe explicitly the behaviours of the information flow.

**Results:**

In this study, we propose new concepts of dissipation, saturation and direction to decipher the information flow behaviours in the pathways and thereby infer the biological pathways from a given source to its target. This model takes into account explicitly the common features of the information transmission and provides a general framework to model the biological pathways. It can incorporate different types of bio-molecular interactions to infer the signal transduction pathways and interpret the expression quantitative trait loci (eQTL) associations. The model is formulated as a linear programming problem and thus is solved efficiently. Experiments on the real data of yeast indicate that the reproduced pathways are highly consistent with the current knowledge.

**Conclusions:**

Our model explicitly treats the biological pathways as information flows with dissipation, saturation and direction. The effective applications suggest that the three new concepts may be valid to describe the organization rules of biological pathways. The deduced linear programming should be a promising tool to infer the various biological pathways from the high-throughput data.

## Background

Pathways play important roles in the biological systems, forming the basis of various biological phenomena, e.g. regulation of gene expression, metabolic pathways, signal transduction and cell cycle control. That how to map the pathways that connect a source and its target from the high-throughput genomic and proteomic data is a challenging but very important question in the post-genome era.

Biological pathways consist of various bio-molecular interactions, e.g. protein-protein interactions, protein-DNA interactions, protein-RNA interactions, RNA-RNA interactions and small-molecule-protein interactions. Since the protein-protein interactions and protein-DNA interactions are available genome-widely for model organisms whereas other types of interactions are very rare, the interactions discussed in the article are limited to protein-protein and protein-DNA interactions.

The pathways include both the endogenous genetic information processing pathways and the exogenous environmental signal transduction pathways. The genetic information processing pathways are mediated by protein-DNA, protein-RNA, RNA-RNA and protein-protein interactions. And the source and target can be identified by the expression quantitative trait loci (eQTL) mapping experiments. In the eQTL mapping studies, the expression levels of genes are treated as quantitative traits and the genetic loci of these phenotypes are mapped by integration of genome-wide genotyping and gene expression profiling [1]. The genetic loci regulate the expression of their target genes through pathways. The environmental signals are generally transmitted to the downstream transcription factors by receptors or second messengers. We only consider the pathways mediated by protein-protein interactions because the second messengers are seldom measured accompanying with the genome-wide gene expression profiling. In this type of study, the source can be a receptor embedded in the plasma membrane and the target a response transcription factor in the nucleus. Recently great interests have been arisen in eQTL mapping and observing of the changes of gene expression after external stimulating [2-5]. These studies bring forward plenty of biological questions, especially how to infer the pathways for a given source and its target. With the development and applications of high-throughput technologies, e.g. yeast two-hybrid systems, ChIP-Chip technology and gene expression microarrays, abundant data of the protein-protein interactions, protein-DNA interactions and gene expression profiles are available. These data provide an opportunity to infer the pathways computationally.

Several methods have been proposed to infer the pathways connecting a specific pair of source and target from the interactions and gene expressions data, e.g. the Color Coding Method [6], Netsearch [7] and the integer linear programming model[8,9]. These methods use different heuristics to find meaningful biological pathways. The Color Coding method assigns a confidence value to each interaction by using logistic regression based on the gene expression and interaction data. It searches the whole network to find paths of a fixed length with the highest score, which is defined as the product of the confidence values assigned to its interactions. Netsearch provides a new statistical method to score the paths of a certain length based on clustering of gene expression data. These two methods both require predefining the pathway structure and pathway length. However, for unknown pathways it is hard to get this type of information in advance. The integer linear programming model proposed by Zhao *et al*. does not require predefining the pathway structure or length[8]. It searches the network to find a subnet with the highest weight sum that connects the source with the target. The weight of each edge is assigned based on the confidence scores of the interactions or the correlation coefficients of genes from gene expression data. This method has a disadvantage that it cannot always guarantee the connectivity of the inferred pathways by confining the degrees of nodes in the pathways. A revised version of the integer linear programming model incorporates the concept of network flow, but it is used only to guarantee the connectivity and control the number of proteins involved in the pathways and the network flows on the edges are not related to the heuristics[9]. Another disadvantage of this method is that it is hard to get the exact solution because the integer linear programming problems are NP-hard. The methods mentioned above use different heuristics to guide the identification of biologically meaningful pathways. But the heuristics are not so straightforward. And all of these methods infer the pathways only from the protein-protein interactions.

The biological pathways are special types of information channels to transmit and process either genetic information or environmental signals in nature. Tu *et al. *use the random walk technique to simulate the information flow and build up a computational model to infer the causal gene and the related pathways underlying the eQTL associations[10]. Suthram *et al. *replace the random walk with the electric current and solve the "dead end" problem of the random walk, improving the accuracy of the pathway inference[11]. These two methods can handle not only the protein-protein interactions but also the protein-DNA interactions. So they can infer the gene regulatory pathways. However, they originally are designed to infer the causal genes at the eQTL of a specific transcript. It still requires other path-searching algorithms to infer the underlying pathways. For example, Suthram *et al. *search the shortest and the most weighted paths and treat them as the biological pathways. More importantly, the irrelevant paths between a source and its target are always assigned positive scores by either the random walk method or the electric current method. This is just like the "dead end" case, which damps the scores of the significant pathway.

In this study, we abstract the problem of the pathway inference as a question to search the paths transmitting the maximal information flow. Dissipation, saturation and direction, the basic attributes of the information flow, are proposed to depict the information flow behaviours in the bio-molecular networks. The whole approach is formulated as a linear programming problem. The results indicated that dissipation naturally educed a path with the highest product of the values assigned to its edges, which was the same as the objective pathway sought by the Color Coding Method [6]. But our method did not require the predefinition of the pathway structure or length. The combination of dissipation and saturation defined a subnetwork between the source and the target. The subnetwork neither was the linear paths sought by the Color Coding Method[5] and Netsearch[7] nor over-scored the irrelevant paths. The direction information of the interactions was easily incorporated by confining the flow directions. Consequently protein-DNA interactions can be incorporated and genetic information pathways can be inferred by our approach. The information flow naturally guarantees the connectivity of the predicted pathways. Experiments on the real data to interpret the yeast eQTLs associations and to infer the yeast MAPK signalling pathways based on interaction and gene expression data showed that the results were consistent with current biological knowledge curated by KEGG [12]. The effectiveness of our method suggests that the information-flow based model with dissipation, saturation and direction may provide an excellent framework to model the biological pathways. The linear-programming formulation makes sure that this method can be solved efficiently and applied to the large interactome. This approach should serve as a promising tool to mine high-throughput genomic datasets.

## Results

### Overview of the information flow model with dissipation, saturation and direction

**Figure 1:**
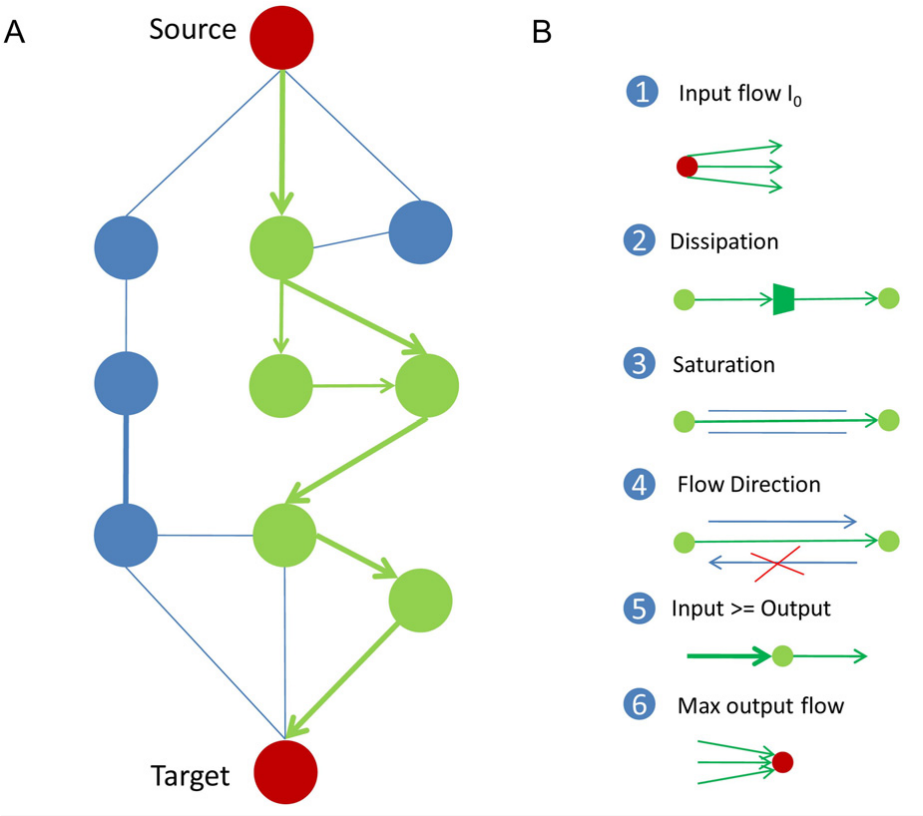
**A schematic diagram of the information-flow model with dissipation, saturation and direction for pathway inference**. A. Simplified example of the pathway inferring problem. The paths composed of green nodes and edges are the inferred pathway whereas the blue nodes and edges are predicted to be not relevant. The edge thickness denotes the capacity limit of each edge. B. Constraints imposed on the edges and the nodes. B1. The source only sends information flows out. The total amount is given by *I*_0_. B2. The information flow dissipates on each edge, illustrated by the thickness of the edge. B3. There is a capacity limit on each edge. B4. The information only flows in the direction of the interactions. B5. The amount of the input flow should not be less than the amount of the output flow at each intermediate node. B6. The target only receives information flows in. The goal is to maximize the total information flow that the target receives.

Our method aims to infer the pathways from the bio-molecular interaction network and gene expression data by maximizing the information flow the target receives with the constraints of dissipation, saturation and direction. Gene expression data contain the dynamic information of cellular responses to various conditions. The bio-molecular network is edge-weighted by calculating the Pearson correlation coefficients based on the gene expression data, the same as the electric current method[11]. Given a source and its target, the objective is to maximize the information flow the target receives. A set of constraints were added according to balance, dissipation, saturation and direction (Figure 1). Balance defines the constraints on the nodes. It requires that the source only sent out the information flow but did not receive, that the target only receives, and that the out flow of each intermediate node is less than or equal to the input flow at the same node. Dissipation, saturation and direction give the constraints on the edges. The information flow decays on each edge according to the dissipation index defined by the edge weight. Each edge has a capacity limit and saturation would occur when an information flow larger than the capacity limit flows through that edge. Some edges with directions only allow the information flow along the specified directions. The capacity limits are introduced because the specific structures, physic-chemical properties and the network-topological positions of the bio-molecules determine the types and amounts of the information they could transmit. The capacity limits cause the saturation and thus the pathways are of forks. We simulated the saturation effect by a stochastic searching method due to the absence of the specific details of the bio-molecules and the interactions. The formulations and details of the model are in the methods section below. We will refer to our method as IFDSD (Information Flow based method with Dissipation, Saturation and Direction) for convenience below.

### Application to the yeast signal transduction pathway inference

The yeast MAPK signal transduction pathways are often used to test the effectiveness of the pathway inference algorithms [5,7-9]. We also applied our model to predict the yeast pathways and compared it to the other methods.

The DIP Core dataset of yeast protein-protein interactions was downloaded at July 8th, 2008 [13]. Only the physical interactions are retained to infer the signalling pathways. Totally 4770 interactions of 2334 proteins were selected. The gene expression data is downloaded from the NCBI GEO database with Accession Number GDS104 [14,15]. It contains the gene expression data of seven time points during the sporulation (0, 30 min, 2 hrs, 5 hrs, 7 hrs, 9 hrs and 11 hrs). A weight was assigned to each interaction by calculating the absolute value of the Pearson correlation coefficient of the two interacting genes. The final weighted interaction network is used at last.

**Figure 2:**
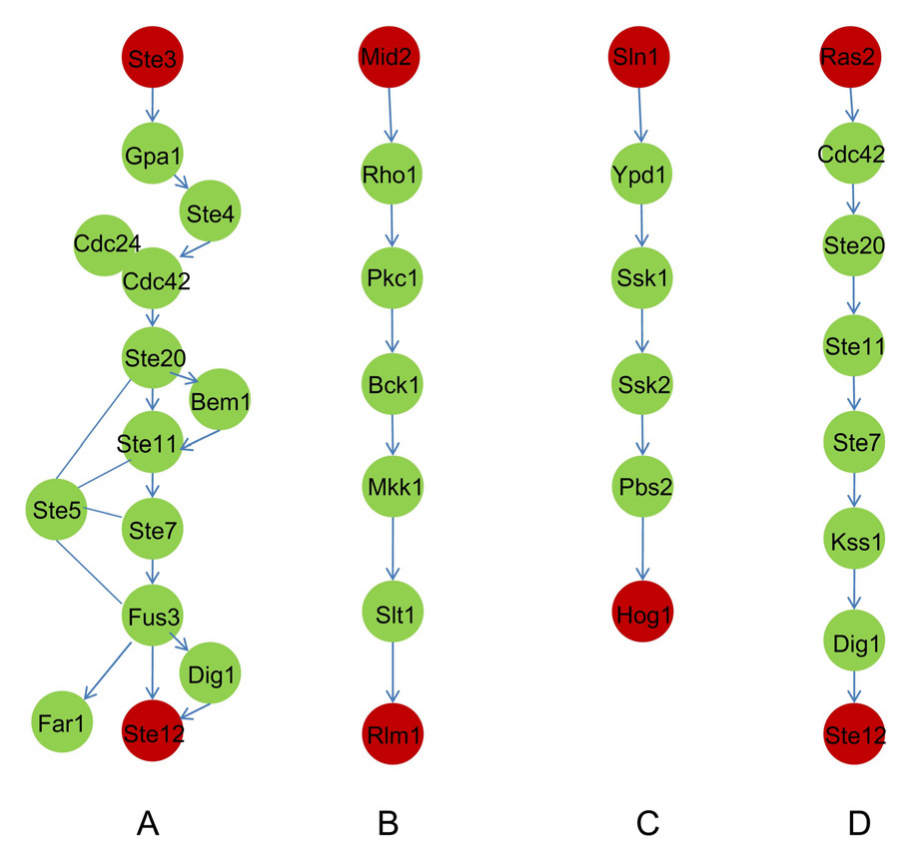
**The yeast MAPK signalling pathways deposited in KEGG **[24]. A. The pheromone-induced yeast MAPK pathway from Ste3 to Ste12. B. The yeast MAPK pathway induced by hypotonic shock from Mid2 to Rlm1. C. The yeast MAPK pathway induced by high osmolarity from Sln1 to Hog1. D. The starvation-induced yeast MAPK pathway from Ras2 to Ste12.

**Figure 3:**
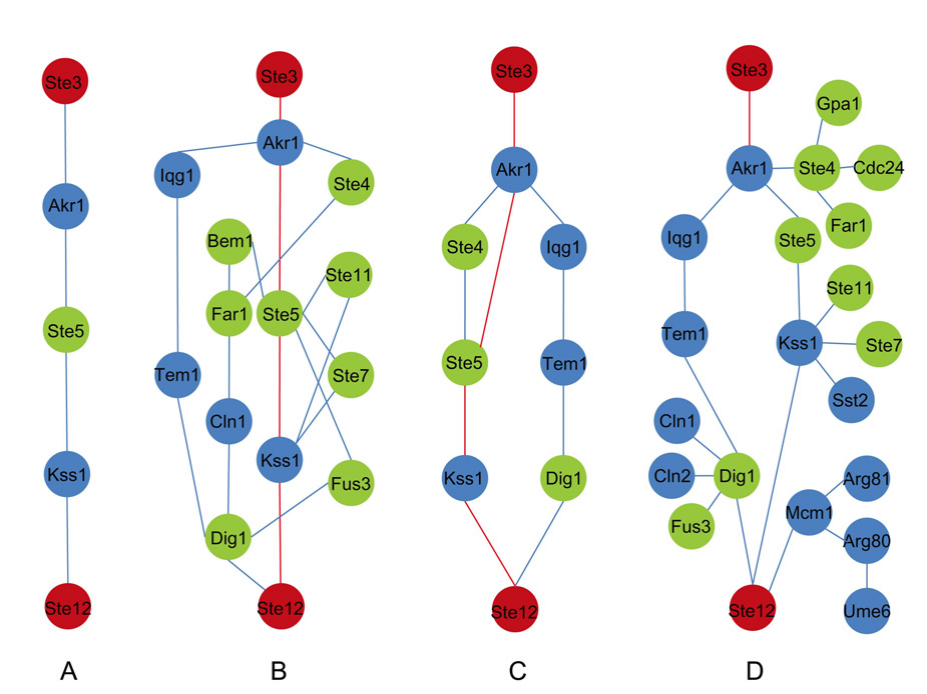
**The predicted yeast MAPK pathways induced by pheromones**. A. The shorted path from Ste3 to Ste12 which is also the result of the Color Coding method with the path length as 5. B. The pathway predicted by the information flow method with dissipation, saturation and direction. C. The pathway predicted by the integer linear programming model[8]. D. The pathway predicted by the electric current method[11]. Red: the source or the target; green: proteins appeared in the yeast pheromone-induced MAPK pathway from Ste3 to Ste12 in KEGG[24]; Blue: false positive proteins.

First, we applied our method to predict the pheromone-induced MAPK signaling pathway (Figure 2A). Given the source protein Ste3 and the target protein Ste12, we applied our approach to the weighted network with *K *= 10, *N *= 5, where *K *and *N *are the parameters of the stochastic searching method (see the methods section for details). The result (Figure 3B) naturally includes the backbone of the MAPK pathway revealed by the Color Coding method (Figure 3A) but here we did not require the prior information of the path length. Based on the backbone, the MAPKKK (Ste11), MAPKK (Ste7) and MAPK (Fus3) were identified sequentially. Eight out of the twelve intermediate proteins in the pheromone-responsive MAPK pathway were predicted accurately. The integer linear programming method proposed by Zhao *et al*.[8,9] (termed as ILP for convenience) can also identify subnet directly. But their heuristic is to find the most weighted subnet in which the degree of each intermediate node is larger than 2. So their method is not sensitive enough to pick out the poorly-weighted interactions (Figure 3C). The electric current based method (termed as EC) was also used to infer the yeast MAPK pathways. Since the EC method also assigned positive weight to the irrelevant edges, the predicted pathway would be as big as the whole network if all the positively-weighted edges were retained. A threshold was set to filter the irrelevant edges. The pathway from Ste3 to Ste12 predicted by the EC method recalled more true positive proteins of the pheromone-induced MAPK pathway but also included more irrelevant proteins (Figure 3D). What's more serious is that most of the "intermediate" proteins had only one edge, which means they no longer were intermediate. But this would not happen in the results of IFDSD and ILP.

Four additional proteins are included in the pathway predicted by IFDSD. AKR1 is included in every predicted pathway because in the used protein-protein interaction data STE3 interacts only with AKR1. IQG1 is an essential protein required for determination of budding pattern. TEM1 is a GTP-binding protein of the Ras superfamily involved in the termination of the M-phase. It controls the dynamics of actomyosin and septin during cytokinesis. CLN1 is a G1/S-specific cyclin protein and plays an important role in cell cycle control. Because the gene expression data we used were generated during the yeast sporulation whereas sporulation and normal cell growth are two mutually exclusive developmental processes, interactions of these proteins with MAPK pathways identified by our method may suggest how MAPK pathways and proteins involved in the control of cell cycle act in concert. But they may also result from the incompleteness and noises of the protein-protein interaction network.

The correlation coefficient of the expression profiles of two genes is often used to weight the edges of bio-molecule networks. Tu *et al. *use the co-expression measure to calculate the probability of random walks[10]. Suthram et al. use the co-expression measure as the electric conductance[11]. In our model we still use this measure to assign the dissipation indices. Intuitively, the efficient information transmission requires the sender and its direct receiver co-exist temporally. And the stoichiometry of the interactions also makes the co-expression requests. The efficiency of information transmission would be poor otherwise. We added a random control to highlight the effectiveness of this weighting scheme by maintaining the whole structure of the original network but assigning the edge weights randomly. The results suggested that the co-expression weighting scheme improved the precision and recall rate significantly (see Additional File 1: Figure S1, p-value = 0.0486).

Another three MAPK pathways of the yeast were also predicted based on the same edge-weighted network and four merits were compared among IFDSD, ILP and EC (Table 1). First, we compared the connectivity of the predicted pathways. IFDSD always generated connected pathways no matter what parameters were selected. However, the connectivity of the pathways predicted by ILP and EC depends on their parameters because their parameters can filter the less-weighted edges (see Additional File 1: Figure S2, S3, S4 and S5). If the parameters are very small, the connectivity of the predicted pathways is guaranteed but the irrelevant edges are included. It decreases the precision and specificity. Otherwise, a increasing precision would harm the connectivity of the pathways. Second, IFDSD and ILP always generated pathways in which the intermediate nodes between the source and the target had two or more edges. But EC can not guarantee that the predicted pathway has this property. By making sure the connectivity of the predicted pathways, we computed the precision and recall rates of the three methods on the four MAPK pathways. Here, the precision means the proportion of true positive proteins in the protein list of the predicted pathways. The recall rate means the proportion of the correctly-predicted proteins in the protein list of the actual pathways. The results suggested that the EC method performed best if we did not consider the connectivity and "intermediate" request. If the "intermediate" condition must be satisfied, the IFDSD method outperforms the ILP method.

**Table 1:** Comparison of IFDSD, ILP and EC on the yeast MAPK pathways.

Source	Target	Method	Connectivity	Intermediate	Precision	Recall
Ste3	Ste12	IFDSD	Yes	Yes	0.67	0.71
		ILP	Depend on λ	Yes	0.56	0.36
		EC	Depend on cutoff	No	0.48	0.79

Ras2	Ste12	IFDSD	Yes	Yes	0.17	0.63
		ILP	Depend on λ	Yes	0.16	0.38
		EC	Depend on cutoff	No	0.16	0.75

Mid2	Rlm1	IFDSD	Yes	Yes	0.27	0.57
		ILP	Depend on λ	Yes	0.18	0.57
		EC	Depend on cutoff	No	0.29	0.71

Sln1	Hog1	IFDSD	Yes	Yes	0.60	1.00
		ILP	Depend on λ	Yes	0.33	1.00
		EC	Depend on cutoff	No	0.86	1.00

Four merits were compared among IFDSD, ILP and EC based on the yeast MAPK pathways. "Connectivity" and "intermediate" were about edges while precision and recall were about the nodes. Pathways predicted by IFDSD are always connected but the connectivity of the pathways predicted by ILP and EC depends on the parameters because they could filter the less-weighted edges. Since the nodes except the source and the target should transfer information from the source to the target, these "intermediate" nodes should have more than two edges linked to them. IFDSD and ILP always generate pathways satisfying this request whereas EC can not. Making sure the connectivity of the predicted pathways, the precision and recall were calculated by selecting the optimal parameters for each method on the yeast MAPK pathways.

### Application to the yeast gene regulatory pathway inference

Unlike the signal transduction pathway that is composed mainly of the interactions of proteins and terminates at transcription factors, pathways mediating the eQTL associations are composed of both protein-protein interactions and protein-DNA interactions. The genetic regulatory information should be transmitted from proteins to the DNA level. To test the performance of our method on this type of pathways, we used it to infer the pathways that mediate the genetic information processing pathways from Gpa1 to Prp39. The genomic locus of Gpa1 is identified to be an eQTL of Prp39 in yeast [16]. GPA1 is analyzed to be the causal gene at that locus [10].

**Figure 4:**
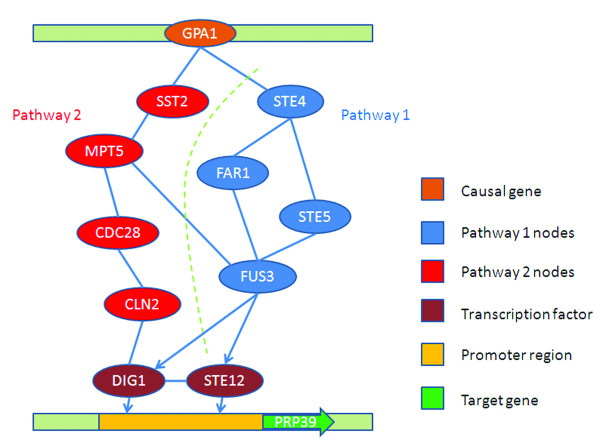
**Pathways between Gpa1 to Prp39 predicted by our method**. Two pathways are inferred. Pathway 1 is the pheromone signaling pathway identified by both Tu *et al.*'s method and the information flow model. Pathway 2 is identified only by the information flow model. It regulates the recovery from the pheromone arrest.

Prp39 is a component of the RNA splicing complex, which is necessary for the stable interaction of mRNA precursors with the snRNP components of the pre-mRNA splicing machinery [17]. Linkage analysis indicates that expression variation of Prp39 is significantly associated to a locus on chromosome VIII and Gpa1 is predicted to be the causal gene at that locus [10]. Given the causal gene Gpa1 and the target gene Prp39, we inferred the pathways mediating the genetic information processing from an integrated network (*K *= 6, *N *= 5), in which the flow direction was confined to be from the protein to the DNA and from the kinase to the substrate (Figure 4). The result showed that Gpa1 may regulate the expression of Prp39 through a pheromone signaling pathway, which is consistent with the result shown by Tu *et al.*[10]. Besides the pheromone signaling pathway, our method also identified an alternative path from Gpa1 to Prp39 (Gpa1-Sst2-Mpt5-Cdc28-Cln2-Dig1-Prp39). Sst2 is a GTPase-activating protein for Gpa1. It regulates desensitization to alpha factor pheromone and is also required to prevent receptor-independent signaling of the mating pathway [18]. Mpt5 is a member of the Puf family of RNA-binding proteins. It binds to mRNAs encoding chromatin modifiers and spindle pole body components and is involved in longevity, maintenance of cell wall integrity, and sensitivity to and recovery from pheromone arrest[19]. Cross-talks of this pathway with the pheromone signaling pathway may suggest the mechanism through which the polar bud growth and the cell cycle are coordinately regulated.

Functional enrichment analysis of the two pathways from Gpa1 to Prp39 indicates that they coordinate to regulate the transitions between pheromone arrest and cell cycle (Table 2), providing an excellent evidence of the effectiveness of our method.

**Table 2:** Functional enrichment analysis for the pathways from GPA1 to PRP39 identified by IFDSD.

Pathways	GO term	Corrected *P*-value	Pathway Frequency	Genome Frequency
Pathway 1:	Pheromone-dependent signal transduction during conjugation with cellular fusion	1.0285e-8	4/4, 100%	29/5819, 0.4%
	Response to pheromone	7.4146e-7	4/4, 100%	101/5819, 1.7%
	Filamentous growth	8.2752e-5	3/4, 75%	105/5819, 1.8%
	Cell cycle arrest	8.2752e-5	2/4, 50%	12/5819, 0.2%

Pathway 2	Adaptation to pheromone during conjugation with cellular fusion	4.2907e-6	3/4, 75%	15/5819, 0.2%
	Negative regulation of signal transduction	2.5475e-5	3/4, 75%	30/5819, 0.5%
	Re-entry into mitotic cell cycle after pheromone arrest	4.1196e-5	2/4, 50%	3/5819, 0.0%
	Negative regulation of cellular process	1.2814e-4	4/4, 100%	290/5819, 4.9%

## Discussion

Pathways play important role in biological systems. How to predict the pathways computationally is a challenging important question in the post-genomic era. Given a biological network, a source and its target, and the gene expression data, previous studies have proposed different heuristics and designed different computational methods to address this question. But it is unclear why these heuristics, e.g. the shortest heuristic[5], the most weighted heuristic[8,9], the random walk heuristic[10] and the electric conductance heuristic[11], are related to the pathway inference. In this study, we introduced dissipation, saturation and direction to describe the behaviours of the information flows in the biological pathways and built a new model. The new method naturally deduced the shortest heuristic [5] but did not require the prior information of pathway structure or pathway length. The predicted pathway was always connected only if the source and its target are in the same connected component in the interactome. Dead ends would no longer influence the predictions compared to the random-walk-based method [10]. And the intermediate nodes in the predicted pathway always have two or more edges because the information flow was local in our model whereas the electric-current-based method would generate intermediate nodes with one edge because the electric current is global [11].

Dissipation and saturation are very common phenomena during the signal transmission in the real world whereas the direction is a basic attribute of information flows. In the biological systems, these concepts should still be effective. In fact, the molecular events underlying cellular processes are subject to random fluctuations [20]. And non-functional interactions of proteins interfere with the formation of functional specific complexes and pathways[21]. The random fluctuations and the non-functional interactions should add noise into the signal and make the signal decay during the transmission process. A series of complicated strategies should be evolved to evade or even overcome the dissipation in the biological systems. The signal transduction cascade may be one of the strategies [22]. A recent study of the biochemical reaction networks reveals the structural sources of the cellular robustness[23]. It should be reasonable and promising to infer the biological pathways by maximizing the information flows between the source and its target with the dissipation constraint.

The saturation phenomenon is obvious in the Internet because everyone wants his/her bandwidth bigger. In the biological systems, it may hide behind the heterogeneous bio-molecules. Each bio-molecule has its specific structure, specific physic and chemical properties, specific interactions with other bio-molecules and specific temporospatial patterns. The specificity of a signalling bio-molecule should determine the type and amount of the information it could convey. For example, an insulin receptor can only bind to the insulin molecules and convey the signal coded in the insulin. But a more advanced cellular order may require the cooperation of many cellular components besides the downstream of the insulin receptor. The other parts of the advanced cellular order would be conveyed through other signalling bio-molecules, which means that saturation happened. Every signal transduction process is tightly regulated. But the regulatory order can not be transferred through the same information channel as the regulated signal. Otherwise the regulation would fail. This should be another example of saturation. A third example may be that a transcription factor has many target genes with different affinities. When the binding sites of the high-affinity target genes were occupied by the specific transcription factor itself or other molecules, it would bind the low-affinity target genes and regulate the transcription of these genes. Due to the stochastic nature of the biological systems, the bindings of high-affinity and low-affinity target genes may be simultaneous, but the trend would exist.

Dissipation, saturation and direction may provide useful concepts to explore the evolutionary achievements of the biological pathways. But predicting pathways correctly depends on the completeness and quality of the bio-molecule networks heavily. Now only the protein-protein interactions and protein-DNA interactions are available genome-widely. The protein-RNA, RNA-RNA, protein-metabolite and other types of interactions are the same important as protein-protein and protein-DNA interactions. The available protein-protein and protein-DNA interactions data are still far from the ultimate real interactome, and the dynamic details of the interactions are unknown.

There are more than thousands of bio-molecules in the biological networks. This brings forward a big challenge on the computational ability. The algorithmic drawback of an effective computational method on small networks would be magnified dramatically. We formulated our model as a linear programming problem because there have been efficient algorithms to solve linear programming problems of thousands of variables and thousands of constraints. It should consider more dynamic details of the biological systems to reach the biological reality in the future when modelling. For example, the enzymatic reactions are described by the Michaelis-Menten kinetics. It would be more accurate to model by the Michaelis-Menten equations than by the linear equations.

## Conclusions

In this study, we proposed a new information flow based model with dissipation, saturation and direction to predict computationally biological pathways from the biological networks. The model was formulated as a linear programming question and applied to infer the yeast MAPK signalling pathways and the genetic regulatory pathways. The results suggest that our method can predict the pathways without the prior information about the pathway structure and pathway length. It can always guarantee the connectivity of the predicted pathways. And it does not generate the false "intermediate" nodes. The precision and recall rates of our method are comparable with the methods that do not satisfy these properties. It can integrate various types of bio-molecular interactions. The effectiveness of our method suggests that dissipation, saturation and direction may provide a useful framework to model the organization of the biological systems. The linear programming model should be a promising tool to mine the huge biological network dataset in the future.

## Methods

### The formulation of the information-flow model with dissipation, saturation and direction

Given a network *G(V,E,D,C,T)*, where *V *is the node set, *E *is the edge set, *D *is the dissipation index set, *C *is the capacity set and *e*_*ij *_∈ *T *denotes the flow direction on *e*_*ij *_is from *i *to *j*. Let *s *be the source and *t *be the target. We define four variables *O*_*ij*_, *O*_*ji*_, *I*_*ij *_and *I*_*ji *_for each edge *e*_*ij*_. *O*_*ij *_denotes the output flow of *i *from *i *to *j*. *I*_*ij *_is the input flow of *j *from *i *to *j*. *O*_*ji *_stands for the output flow of *j *from *j *to *i *and *I*_*ji *_is the input flow of *i *from *j *to *i*. The approach is formulated as a linear programming model:(1)

Subject to(2)(3)(4)(5)(6)(7)(8)(9)(10)

where *I*_0 _is the total amount of the output flow at the source node. *C*_*ij *_represents the capacities on the edge *e*_*ij *_. *D*_*ij *_denotes the dissipation index on the edge *e*_*ij *_and *D*_*ij *_refers to the set of interactions with directions, especially protein-DNA interactions. Formulation (1) illustrates that the objective is to maximize the received input flow at the target node. Equations (2) and (3) determine that the source node only sends information. And equation (4) ascertains that the target node does not send out any information flow. Equation (5) shows that the amount of input flow has to be larger than or equal to the amount of output flow at each internal node (nodes except the source and target). Inequalities (6) and (7) require the flow to be nonnegative. Equation (8) defines that the flow from *i *to *j *is dissipated, that is, only part of the output flow *O*_*ij *_at *i *was converted into the input flow *I*_*ij *_at *j *according to the dissipation index *D*_*ij*_. Inequality (9) confines the output flow on each edge not exceeding the capacity limit of that edge. Equation (10) restricts the flow only along the direction of the edge. The reverse flow should be zero.

### The stochastic searching algorithm to simulate the saturation effect

The linear programming model (1)-(10) infers the pathways given the source, the target and the whole network *G(V,E,D,C,T)*, where *V *is the set of proteins and DNAs, *E *is the set of protein-protein interactions and protein-DNA interactions, *D *defines the dissipation indix on each edge, *C *defines the capacity of each edge, and *T *defines the orientations of the interactions. *V*, *E *and *T *can be easily constructed from the large-scale protein-protein and protein-DNA interactions. *D *is defined by the absolute value of correlation coefficients determined by using the expression values of genes [6,8,10,11]. *C *can not be assigned easily because now there is no sufficient experimental information available. We design a stochastic searching algorithm in this study to bypass the assignment problem of *C *in practice. The algorithm is described as follows:

1. For *k *= 1, set *C*_1 _large enough for each edge (e.g. *I*_0_), solve the linear programming model (1)-(10) with parameters *G(V,E,D,C_1_,T) *and get the solution *X*_1 _. *X*_1 _is a simple path from the source to the target.

2. For *k *= *i*(*i *> 1), randomly select one of the edges of *X*_*i*-1 _and denote the selected edge as *p*. Let *C*_*i *_= *C*_*i*-1_, set the capacity of *p *as zero and update *C*_*i*_. Solve the linear programming model (1)-(10) with parameters G(V,E,D,C_*i*_,T) and get the solution *X*_*i*_.

3. Repeat (2) until *k *reaches the allowable times *K*.

4. *X*_*i*_L *X*_*k *_are all simple paths. Assemble *X*_*i*_L *X*_*k *_will get a subnet connecting the source and the target. Set the subnet as the last solution to the original problem defined by (1)-(10) in which *C *is unknown.

The idea behind the algorithm is to search the optimal path at first, then to search the suboptimal paths after blocking the optimal path, and repeat this procedure. Saturation is simulated through blocking the available paths. This algorithm is likely to identify the *k*th optimal path from the source to the target. The difference lies in the simulations of saturation through blocking.

Due to the stochastic nature of the algorithm, it will run several times, e.g. *N *times, and then half of the solutions with the higher objective values are selected as the candidate pathways from the source to the target.

There are overall three parameters in this searching algorithm. The first parameter is *I*_0_, which represents the amount of information flow the source sends out. The result is independent of the value of *I*_0_, as long as it is positive. In this study we set *I*_0 _to be 1. The second parameter is *K*, the number of zeros in *C*, which measures the complexity of the inferred pathways. The larger *K *is, the more complicated is the predicted pathway (see Additional File 1: Figure S6). A pathway predicted with a smaller *K *is more significant. The pathway predicted with lager *K *is more complete and includes the pathway predicted with the smaller *K*. The third parameter is *N*, which represents the number of repetitions to counteract the random effect in the stochastic search. The larger *N *is, the robuster the prediction is. *N *is positively related to *K*. The larger *K *is, the larger *N *should be. Experiments showed that the solution became quickly stable when *N *got larger e.g. 5 in the MAPK pathway inferring where *K *= 10 (see Additional File 1: Figure S7). When this method is applied to reveal the underlying pathways between the given source and its target, a small *K*, e.g. 5, and a small *N*, e.g. 5, should be first tested to reveal the most significant parts of the pathway.

### Significance measurement

To measure the reliability of our method, we compare the predictions with current knowledge of the yeast MAPK pathways curated in KEGG [12,24] and the predicted pathways by Tu *et al. *from GPA1 to PRP39 [10]. As the pathways known so far are still incomplete, we further test the consistence of the gene functions of the predicted pathways using the biological process annotations in Gene Ontology (GO) [25]. Gene ontology terms can generally reflect whether genes belong to the same biological processes. The probability that genes of the inferred pathways have the same function is calculated by a hypergeometric distribution implemented in BinGO [26].

## Authors' contributions

XR conceived the information flow model, prepared the data, formulized the linear programming model, analyzed the results and drafted the manuscript. XZ conceived the information flow model and drafted the manuscript. LYW conceived the information flow model and formulized the model as a linear programming problem. XSZ provided critical comments on the linear programming model and the draft. All authors read and approved the final manuscript.

## Supplementary Material

Additional file 1 **Supplementary Figures**. This file contains the supplementary figures which further illustrate the properties of our method and the previous methods. Click here for file
