# Supplementary material for: An information-flow-based model with dissipation, saturation and direction for active pathway inference
Source: BMC Syst Biol. 2010 May 27;4:72. doi: 10.1186/1752-0509-4-72 (PMC2890502; doi:10.1186/1752-0509-4-72)
Supplement: Additional file 1 — Supplementary Figures. This file contains the supplementary figures which further illustrate the properties of our method and the previous methods. [file 1752-0509-4-72-S1.PDF]

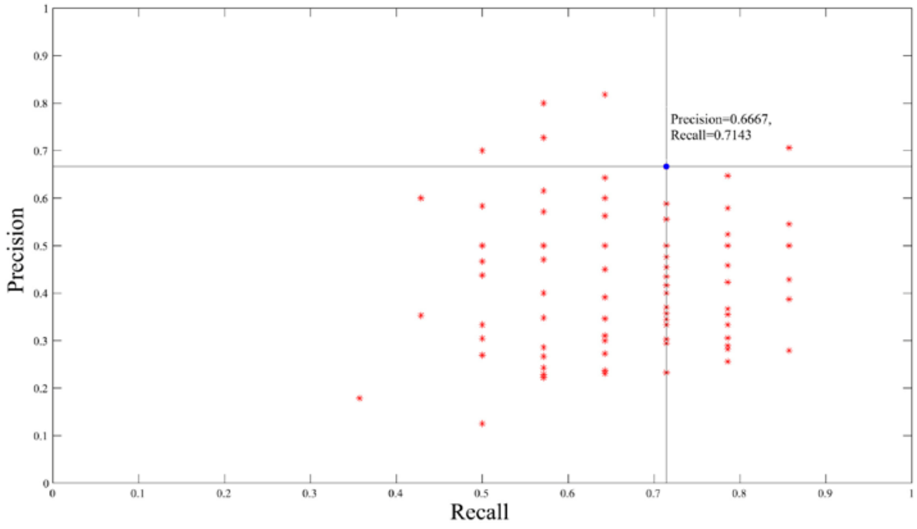

Figure S1 Comparison of the co-expression weighting scheme and the random scheme

The red points are the random instances and the blue point is the co-expression instance. Given the network, random dissipation indices between zero and one were assigned to each edge. Then, the pathway between Ste3 and Ste12 was predicted by our method (*K*=10, *N*=5). This process was repeated for 100 times. The precision and recall rates of these random instances were plotted in Figure S1. The products of the precision and recall rates were used to calculate the p-value.

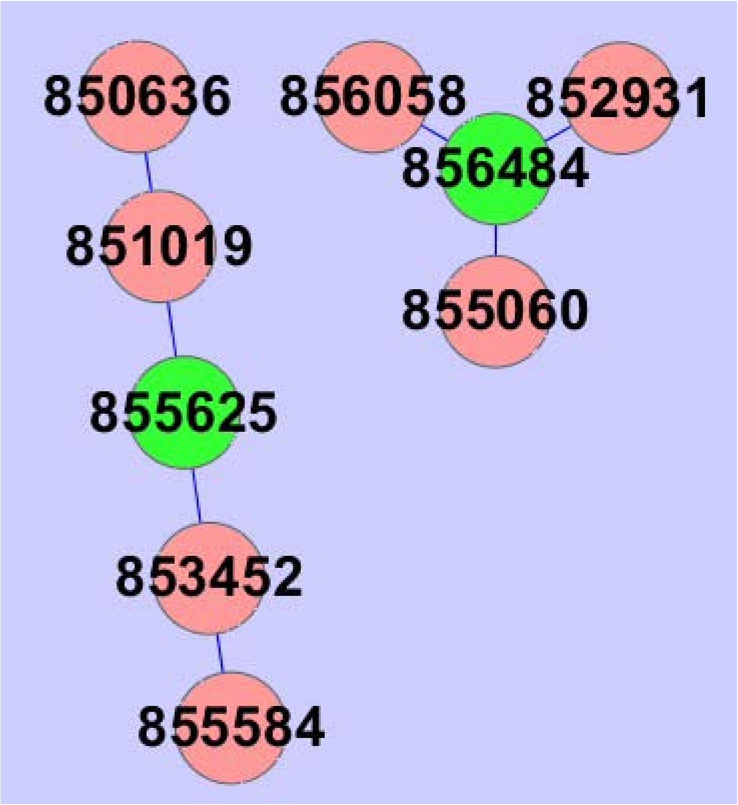

Figure S2 – The pathway from Ras2 to Ste12 predicted by the electric current based method with the threshold 0.1

The paths were calculated by solving the electric current model [1], given the source Ras2 (Entrez Gene ID: 855625, green) and the target Ste12 (Entrez Gene ID: 856484, green). The edges with current less than 0.1were filtered. Obviously, the source Ras2 and the target Ste12 were not connected. The figure was created by Cytoscape[2].

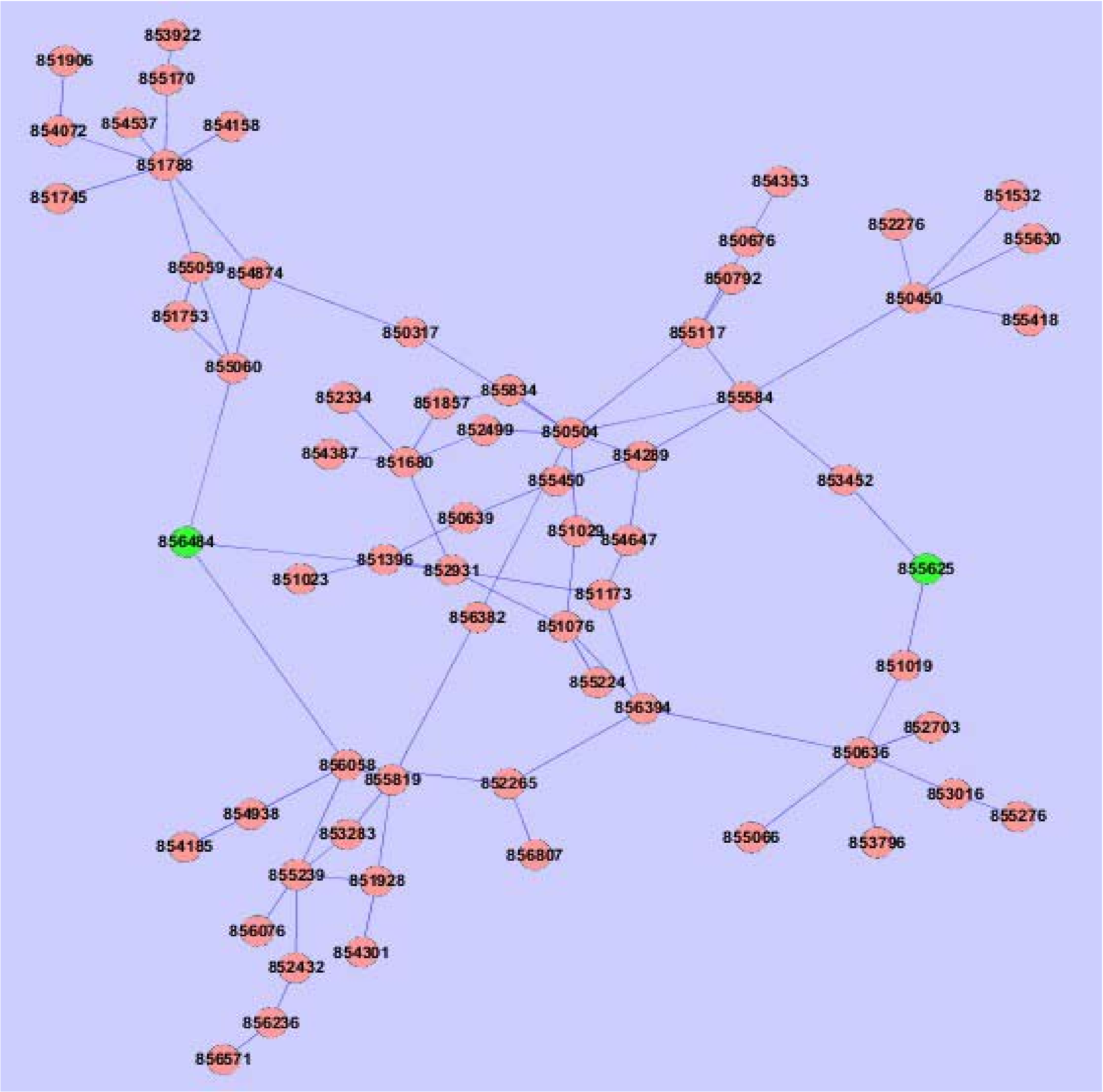

Figure S3 – The pathway from Ras2 to Ste12 predicted by the electric current based method with the threshold 0.01

The paths were calculated by solving the electric current model [1], given the source Ras2 (Entrez Gene ID: 855625, green) and the target Ste12 (Entrez Gene ID: 856484, green). The edges with current less than 0.01were filtered. The source Ras2 and the target Ste12 were connected. The figure was created by Cytoscape[2].

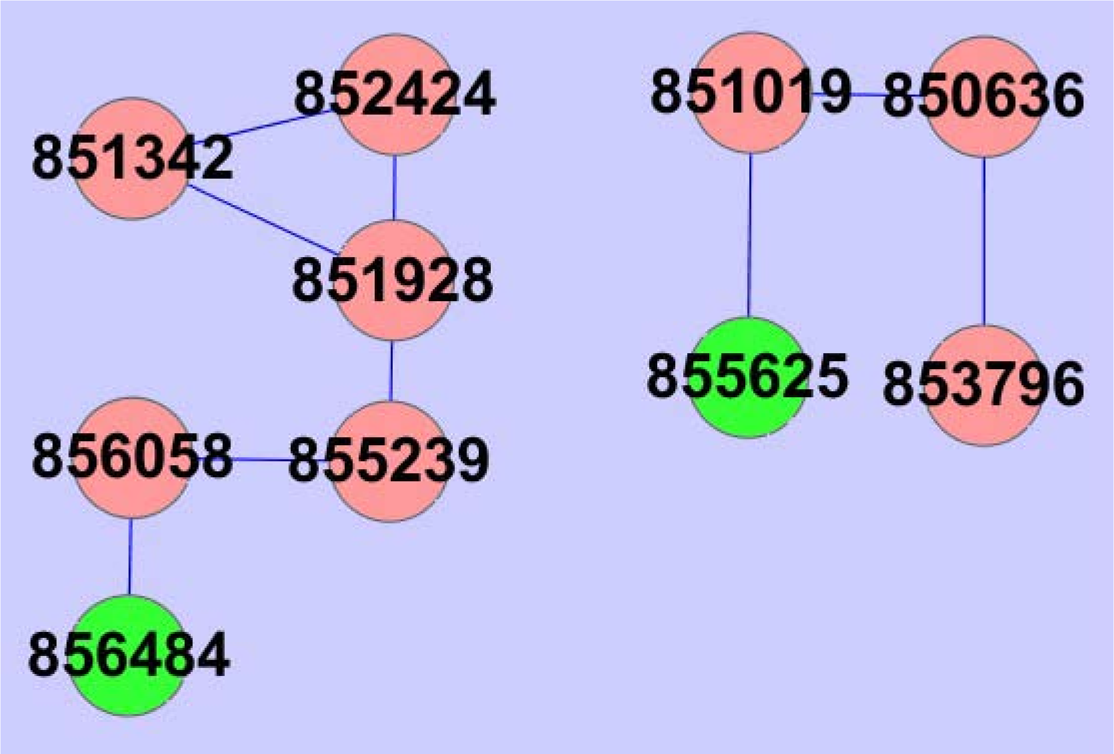

Figure S4 – The pathway from Ras2 to Ste12 predicted by the ILP method with λ =0. 9

The paths were calculated by solving the ILP model [3] with λ =0.9, given the source Ras2 (Entrez Gene ID: 855625, green) and the target Ste12 (Entrez Gene ID: 856484, green). Obviously, the source Ras2 and the target Ste12 were not connected. The figure was created by Cytoscape[2].

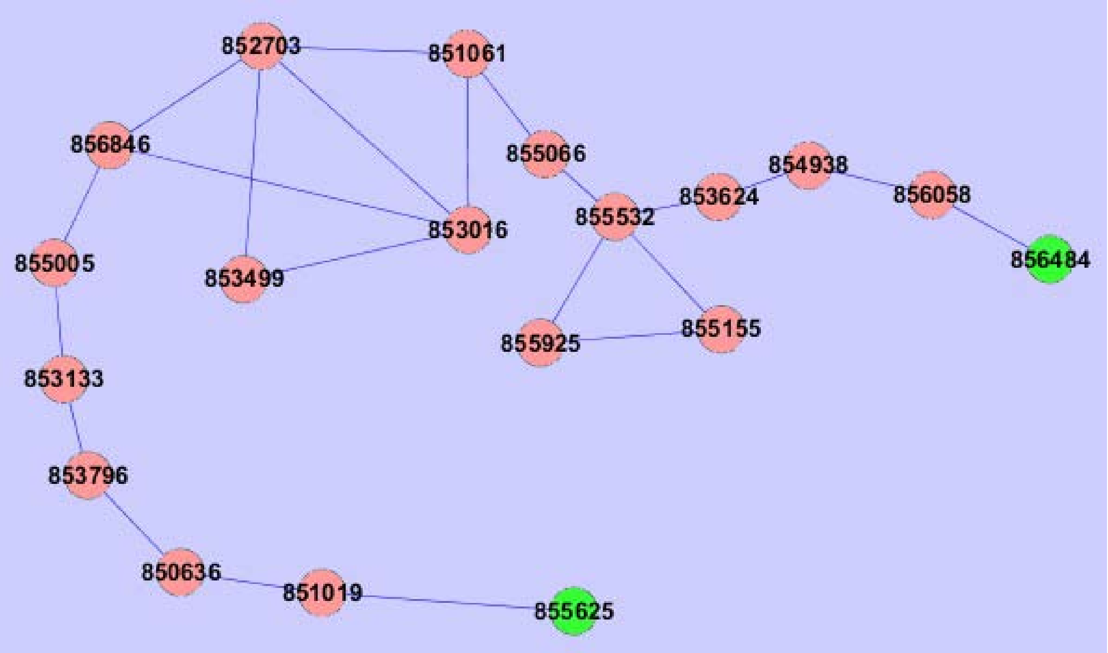

Figure S5 – The pathway from Ras2 to Ste12 predicted by the ILP method with λ =0. 8

The paths were calculated by solving the ILP model [3] with λ =0.8, given the source Ras2 (Entrez Gene ID: 855625, green) and the target Ste12 (Entrez Gene ID: 856484, green). The source Ras2 and the target Ste12 were connected. The figure was created by Cytoscape[2].

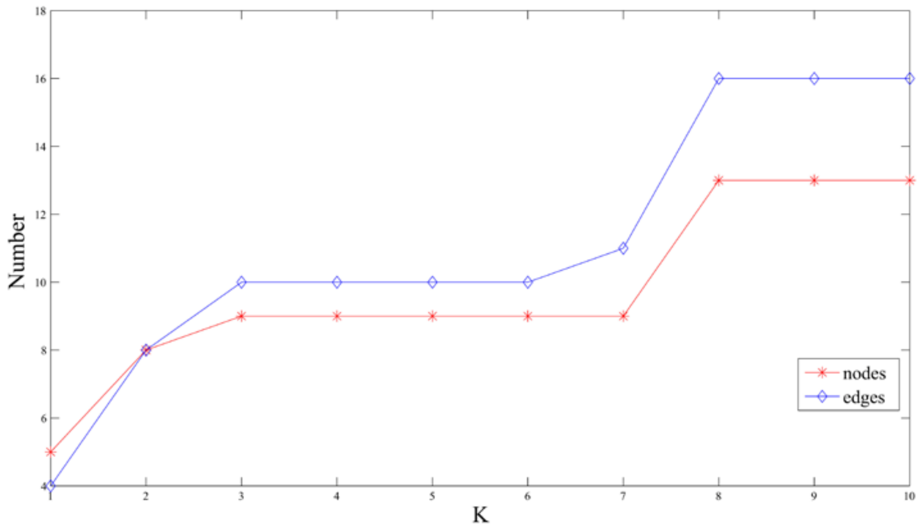

Figure S6 – The numbers of nodes and edges of the pathway from Ste3 to Ste12 increase with the parameter *K*

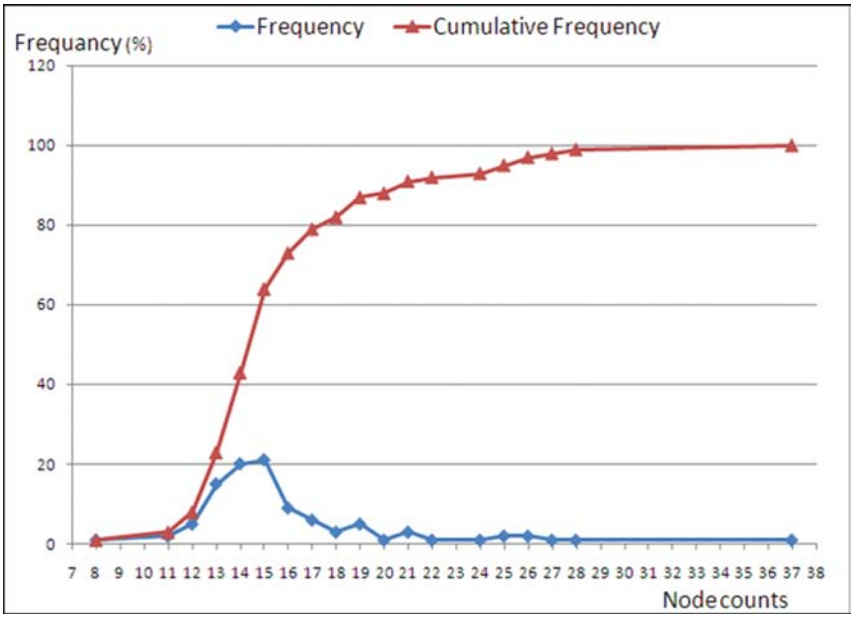

Figure S7 – The distribution of node counts of one hundred predictions of the pathway from Ste3 to Ste12 with *K*=10 and *N*=5

Since our method is a stochastic method, we predicted the pathway from Ste3 to Ste12 with *K*=10 and *N*=5 for one hundred times. The results suggested the robustness of the solutions. Most of the predicted pathways were of thirteen to sixteen nodes.

1. Suthram S, Beyer A, Karp RM, Eldar Y, Ideker T: **eQED: an efficient method for interpreting eQTL associations using protein networks.** *Mol Syst Biol* 2008, **4**.

2. Shannon P, Markiel A, Ozier O, Baliga NS, Wang JT, Ramage D, Amin N, Schwikowski B, Ideker T: **Cytoscape: A Software Environment for Integrated Models of Biomolecular Interaction Networks.** *Genome Research* 2003, **13:**2498-2504.

3. Zhao X-M, Wang R-S, Chen L, Aihara K: **Uncovering signal transduction networks from high-throughput data by integer linear programming.** *Nucl Acids Res* 2008, **36:**e48-.
